# Functional Outcomes of Childhood Selective Dorsal Rhizotomy 20 to 28 Years Later

**DOI:** 10.7759/cureus.1256

**Published:** 2017-05-17

**Authors:** TS Park, Jenny L Liu, Caleb Edwards, Deanna M Walter, Matthew B Dobbs

**Affiliations:** 1 Neurological Surgery, Washington University School of Medicine, St. Louis Children's Hospital; 2 Pediatric Neurosurgery, Washington University School of Medicine, St. Louis Children's Hospital; 3 Pediatric Orthopedic Surgery, Washington University School of Medicine, St. Louis Children's Hospital

**Keywords:** adults, quality of life, cerebral palsy, selective dorsal rhizotomy, ambulation, spasticity

## Abstract

**Background:**

Selective dorsal rhizotomy (SDR) is a surgical method used to treat childhood spastic cerebral palsy (CP). However, the effects of early SDR on functional outcomes and quality of life decades later in adulthood remains to be elucidated.

**Objectives:**

To evaluate the long-term outcomes in terms of satisfaction and mobility of adult patients who received childhood SDR.

**Methods:**

Adult patients who received SDR in childhood were surveyed. The survey questionnaire asked about demographic information, quality of life, health outcomes, SDR surgical outcomes, ambulation, manual ability, pain, braces/orthotics, post-SDR treatment, living situation, education level, and work status.

**Results:**

Our study included 95 patients. The age that patients received SDR was between two and 18 years. The age at the time of survey was between 23 and 37 years (mean ± S.D., 30.2 ± 3.6 years). Post-SDR follow-up ranged from 20 to 28 years (mean ± S.D., 24.3 ± 2.2 years). Seventy-nine percent of patients had spastic diplegia, 20% had spastic quadriplegia, and one percent had spastic triplegia. Ninety-one percent of patients felt that SDR impacted positively the quality of life and two percent felt that the surgery impacted negatively the quality of life after SDR. Compared to pre-operative ambulatory function, 42% reported higher level of ambulation and 42% ambulated in the same level. Eighty-eight percent of patients would recommend the procedure to others and two percent would not. Thirty-eight percent reported pain, mostly in the back and lower limbs, with mean pain level 4.2 ± 2.3 on the Numeric Pain Rating Scale (NPRS). Decreased sensation in patchy areas of the lower limbs that did not affect daily life was reported by eight percent of patients. Scoliosis was diagnosed in 31%. The severity of scoliosis is unknown. Only three percent of them underwent spinal fusion. Fifty-seven percent of patients required some orthopedic surgery after SDR. The soft-tissue tendon lengthening procedures included lengthening on hamstrings, Achilles tendons or adductors. Out of all bone procedures, 24% of patients had hip surgery, five percent had knee surgery, and 10% had derotational osteotomies. No late side effects of SDR surgery were reported in this survey.

**Conclusions:**

In our 95 adult patients who received SDR in childhood, the surgery had positive effects on the quality of life and ambulation 20-28 years later. There were no late complications of SDR surgery.

## Introduction

Selective dorsal rhizotomy (SDR) can significantly reduce or permanently eliminate cerebral palsy (CP) spasticity and improve the mobility of patients with spastic CP. Numerous outcome studies of SDR found improved motor function during short term follow-up [1-4]. However, as patients usually receive SDR during childhood, most follow-up studies have assessed outcomes in children and adolescents [4-7].

Previous quality of life and functional outcomes studies have reported long-term benefits of SDR in CP patients. In a study on 88 adolescents and adults, Hurvitz, et al. found positive effects of childhood SDR on functional outcomes [8]. Our group also recently reported outcomes of 294 adults who received childhood SDR two to 28 years ago [9].

Herein we describe the functional outcomes of patients who have reached adult age after childhood SDR and were followed-up 20 to 28 years later. The objective of this report is to address a pressing question in the SDR literature: What are the long-term outcomes of childhood SDR in patients more than 20 years later? To answer this question, we examined the functional outcomes in 95 patients, who received SDR 20 to 28 years ago, out of the original 294 total patients in our previous study [9].

## Materials and methods

The institutional review board of Washington University School of Medicine granted approval for the present study. We obtained consent from either patients directly or from guardians. The subjects for this study were adults who received SDR in childhood 20 to 28 years ago from 1987 to 1996 at University of Virginia Hospital or St. Louis Children's Hospital. The current study cohort was selected as long-term follow-up patients from our larger study examining outcomes in patients who received childhood SDR two to 28 years ago [9]. All surgeries were performed by the first author.

We gathered contact information from emails, mailing addresses, and phone numbers recorded in our clinic's database and medical records. The study survey was sent out to the potential participants both electronically and via letters in the mail.

Survey questions asked about demographic information, quality of life, health outcomes, surgical outcomes, motor function, manual ability, pain issues, braces/orthotics, post-SDR treatment, living situation, education level, work status, and side effects of SDR.

Demographic survey questions asked for living situation, highest education level, and current employment status. Participants were asked if they experienced urinary incontinence. Patients experiencing sensation loss were further questioned to see if the loss of sensation was complete, partial, and if it caused problems with daily life. Respondents also provided information about any additional spine problems, particularly scoliosis, and if they took any steps to treat these problems.

The Diener Satisfaction with Life Scale (SWLS) was used to assess quality of life and subjective well-being by rating five statements on a scale from one (strongly disagree) to seven (strongly agree). The summary scores on the Diener SWLS were interpreted as followed: (31-35) equaling extremely satisfied with life, (26-30) very satisfied, (21-25) slightly satisfied, (20) neutral, (15-19) slightly dissatisfied, (10-14) very dissatisfied, and (5-9) extremely dissatisfied. The reliability of this scale has been reported to be greater than 0.80 [10].

A five point scale from poor to excellent, as seen on question one of the 36-item Short Form Health Survey (SF-36) [11], was used to assess the perception of one’s personal health. Other questions inquired if patients felt the surgery was beneficial and if they would recommend it for other children. Additional yes-no questions determined if patients needed assistance on certain activities of daily life such as eating, bathing/showering, using the toilet (and if catheterization was needed to empty the bladder), getting dressed, grooming/hygiene, and transferring positions. The participants were also asked yes-no questions about if they regularly strengthened muscles, stretched hamstrings and heel cords, or played a recreational sport.

The patients used the Gross Motor Function Classification System (GMFCS), a scale to measure gross motor function in children with CP [12]. The scale focuses primarily on functional agility related to sitting, walking, and wheeled mobility and has been validated with good correlation between self-reported and professionally determined levels. The five level ambulation classification system is as follows: I-walks without limitation, II-walks with limitation, III-walks using handheld mobility device, IV-limited self-mobility (can sit independently but is unable to stand or walk without significant support), V-transported in manual wheelchairs with extremely limited self-mobility.

To determine the breakdown of ambulation levels in our patient population, we compared pre-surgery mobility levels with survey-reported GMFCS ratings. The patients were also asked to indicate if ambulation had improved, stayed the same, or worsened after SDR surgery.

Using the Manual Ability Classification Scale (MACS) [13], we assessed upper extremity function. Though the MACS was originally validated in children with cerebral palsy, it has been found to have good reliability and validity in CP adults [14]. The scale breaks down as follows: I-handles objects easily and successfully, II-handles most objects with slightly reduced quality, III-handles objects with difficulty, IV-handles limited number of objects, V-does not handle objects. This five level scale measures a patient's ability to handle objects in daily life.

Using the Numeric Pain Rating Scale (NPRS), the patients were asked to report pain experienced within the last week. The NPRS is a zero to 10 scale with zero indicating no pain and 10 being the worst pain imaginable. The participants were also questioned about the location of the pain, and if the patients experienced back pain, they were asked about the location and intensity of back pain using the NPRS. These patients were further questioned about whether they received physician care, medications, surgery, physical therapy, or injections for their back pain. The respondents were also asked if they had constant pain in their legs and if this pain was due to muscle/joint problems or nerve pain.

## Results

A total of 431 patients received childhood SDR 20-28 years earlier between 1987 and 1996. One hundred fifteen patients lacked reliable contact information. A survey questionnaire was sent via email or mail to the remaining 316 patients who were regarded as potential study subjects. Of the 316 patients, 95 (30%) patients responded and participated in the survey. It is of note that the 95 patients were included in a cohort of 294 adult patients in our recent report that focused on functional outcomes in adulthood [9].

### Demographic summary

The patients underwent SDR between 2.0 and 17.9 years of age (mean 6.0 ± 3.5 years). The follow-up period was between 20.1 and 28.3 years (mean 24.3 ± 2.2 years). The age of the patients ranged from 23.4 to 37.2 years (mean 30.2 ± 3.6 years). The subtype of CP was spastic diplegia in 79%, spastic quadriplegia in 20%, and spastic triplegia in one percent (Table 1).

**Table 1:** Demographic Summary of 95 Adults Who Underwent SDR 20-28 Years Earlier

Study Population		Value
Total number of patients		95
Age at surgery	2.0 – 17.9 years (mean 6.0 ± 3.5 years)	
Age at follow-up survey	23.4 – 37.2 years (mean 30.2 ± 3.6 years)	
Follow-up period	20.1 – 28.3 Years (mean 24.3 ± 2.2 years)	
Sex		% of total patients
Male		55
Female		45
Cerebral palsy diagnosis		% of total patients
Diplegia		79
Quadriplegia		20
Triplegia		1

### Gross Motor Function Classification Scale (GMFCS) and Manual Ability Classification Scale (MACS)

At the time of survey, 80% had GMFCS I, ll, and III, and 19% had GMFCS levels IV and V. Eighty-eight percent reported to handle most objects independently with MACS levels of I and II (Table 2).

**Table 2:** Patient Mobility, Activities and Health 20-28 Years After SDR in 95 Patients

Gross Motor Function Classification Scale (GMFCS)	% of total patients
I	21
II	28
III	31
IV	14
V	5
N/A	1
Manual Ability Classification Scale (MACS)	% of total patients
I	69
II	19
III	8
IV	1
V	2
N/A	1
Independent activities of Daily Living	% of total patients
eating	87
bathing	67
toileting	76
dressing	67
hygiene	67
transfers	77
Physical Activities of Daily Living	% of total patients
regularly strengthen muscles at least once a week	60
regularly stretch hamstrings	43
regularly stretch heel cords	36
play recreational sports	22

### Changes in ambulation

At the time of survey, 42% of the patients had improved their level of ambulation since surgery; 42% were ambulating in a level similar to their ambulation level before surgery; 14% experienced their ambulation worsening over time (Table 3).

**Table 3:** Changes of Ambulation Level 20-28 Years After SDR

Ambulation at the time of survey compared to pre-SDR ambulation	% of total patients
improved level of ambulation	42
same level of ambulation	42
worsened level of ambulation	14
no report of ambulation	2

Prior to surgery, 30% ambulated independently in protected or all environments, four percent ambulated with crutches/canes, and 44% ambulated with walkers. Twenty-two percent of the patients were non-ambulatory (Table 4). In patients who reported ambulation 20-28 years later, more patients walked independently (40%) or with crutches/canes (19%). Fewer patients ambulated using walkers (22%), though a portion were wheelchair bound (19%) (Table 4). Twenty-eight percent indicated that they were able to run.

**Table 4:** Pre- and Postoperative Ambulation Levels 20-28 Years After SDR in 95 Patients

Ambulation Level	PRE-SDR % of total patients	POST-SDR % of total patients
Independent walking	30	40
Walking with crutches or canes	4	19
Walking with walker	44	22
Non-ambulatory	22	19

### Patient’s perception of SDR

Ninety-one percent felt that the SDR was beneficial to them, while two percent considered SDR to have decreased their quality of life. Overall, 88% would recommend SDR to other children with CP, two percent (two patients) would not recommend the procedure, and 10% were unsure (Table 5). For the two patients who would not recommend SDR, both patients had spastic quadriplegia. One patient ambulated using a walker, and the other ambulated somewhat independently prior to surgery. After surgery, both could not stand or walk without significant support, requiring a wheelchair to get around.

**Table 5:** Patients’ Perceptions of SDR and Health 20-28 Years After SDR in 95 Patients

How did SDR affect your Quality of Life?	% of total patients
Increased	91
Decreased	2
No change	0
Not sure	7
Would you recommend SDR to others?	% of total patients
Yes	88
No	2
Not sure	10

### Pain

Thirty-eight percent of the patients reported pain mostly located in the back and lower limbs. The mean pain level was 4.2 ± 2.3 on the Numerical Pain Rating Scale (NPRS) (Table 6). 

Constant leg pain was reported by nine percent of the patients. The majority of these patients felt that the pain was due to muscle and joint problems. One respondent reported that the leg pain was diagnosed as nerve pain, and one respondent stated that the leg pain was attributed to both muscle-joint problems and nerve pain (Table 6).

### Urinary incontinence

Urinary incontinence affected 11% of the patients. None of them needed intermittent catheterization to empty their bladder, suggesting that incontinence was not caused by SDR (Table 6).

### Sensory changes

Decreased sensation in the lower limbs was reported by eight percent of the patients. None had complete loss of sensation in their legs and most only had numbness in parts of their legs. The sensory change did not impact their daily life (Table 6).

**Table 6:** Pain, Bladder Function & Sensory Changes 20-28 Years After SDR in 95 Patients

Parameter	% of total patients
Patients experiencing pain	38
	Numerical Rating Scale (0-10)
Average pain score	4.2 + 2.3
Where is the pain located?	% of patients with pain
Back	29
Upper limb	1
Lower limb	16
Head	5
Other	4
	% of total patients
Constant leg pain	9
Cause of pain?	% of leg pain patients
Muscle and joint problem	80
Nerve pain	20
	% of total patients
Urinary incontinence	11
Requiring catheterization	0
Decreased sensation in areas of lower limbs	8

### Post-SDR treatments

Scoliosis was reported by 31% of all patients. Thirty percent of all scoliosis patients pursued some form of treatment. Only three percent of the patients underwent spine fusion surgery. Orthopedic surgeries were carried out in 57% of all patients. The most common orthopedic surgeries were hamstring lengthening (33%), Achilles tendon lengthening (21%), and adductor release (15%). Twenty-four percent of all patients also later underwent hip surgery, 10% of the total patients received derotational osteotomies, and five percent had surgeries on their knees (Table 7).

Three percent of patients (two quadriplegic patients and one diplegia patient) had baclofen pumps (Medtronic, MN, USA) inserted after SDR. Only one quadriplegic patient still had baclofen pump implants at the time of survey. Twenty-two percent of the patients were taking antispasticity medications (Table 7).

**Table 7:** Surgical and Medical Treatments 20-28 Years After SDR in 95 Patients

	% of total patients
Scoliosis and other back issues	31
	% of scoliosis patients
Back issue intervention for scoliosis	30
	% of total patients
Spine fusion surgery	3
Orthopedic surgery	57
Hip surgery	24
Knee surgery	5
Tendon lengthening surgery	50
Hamstrings	33
Achilles tendon	21
Adductors	15
Calf muscles	5
Derotational osteotomy	10
Baclofen pump implanted	3
Currently implanted	1
Currently use oral spasticity medication	22
Currently use lower limb orthotics	34

### Living situation

At the time of survey, 55% lived with their parents and 22% lived alone. Another 22% either lived with a significant other, caregiver, or other adult. For education, 94% completed at minimum a high school level degree and 54% had advanced degrees. Fifty-nine percent of patients were employed and of the employed patients, 54% worked full-time and 44% worked part-time (Table 8).

### Satisfaction with life

Ninety-five percent rated their health as good or better, four percent as fair, and one percent as poor (Table 8). The participants typically gave responses on the higher end of the slightly satisfied bracket of the Diener Satisfaction with Life Scale (SWLS). The mean SWLS score was 25 ± 9.

**Table 8:** Education, Employment and Health Perception 20-28 Years After SDR in 95 Patients

Living situation	% of total patients
With parents	55
Alone	22
With roommate	1
With significant other	13
With caregiver	6
Other	3
Education	% of total patients
Still in school	11
High school diploma/GED	40
Advanced degree (beyond high school)	54
Employment	% of total patients
Employed	59
	% of employed patients
Full-time	54
Part-time	44
N/A	2
Perception of health	% of total patients
Excellent	22
Very good	34
Good	39
Fair	4
Poor	1

## Discussion

In the present study, we examined the functional outcomes of 95 patients 20-28 years after childhood SDR for spastic CP. The long follow-up period in a series of 95 patients is unprecedented among SDR outcome studies reported to date.

The primary goal of SDR at our center is improving a patient's ambulation. Accordingly, the results of our study focused on ambulation levels before and after SDR. Forty-two percent of patients reported an improved ambulation level and 42% reported a similar ambulation level at the time of survey. Our study did not include an assessment of the qualitative changes of gait, such as stride, cadence, balance, and overall gait pattern. These gait parameters regularly improve after SDR.

Untreated spastic CP patients develop decreased strength, lower endurance, and muscle/joint pain that start in late childhood and early adulthood. The "early aging" is caused by spasticity [15]. In our experience, if spasticity is untreated, patients continue to deteriorate and lose the ability to walk after 50 years of age. We have not seen any patient whose CP spasticity resolved spontaneously as they age. Thus the improved or stable ambulation level in 84% of our patients 20-28 years later represents positive outcomes that can be attributed to SDR. Without SDR intervention, many of the patients in this study would have likely deteriorated in ambulation or even required wheelchairs to get around.

At the time of follow-up, 14% of the patients experienced worse ambulation compared to preoperative ambulation. None of these patients ambulated independently or with crutches before SDR. The patients who ambulate with walkers or require greater assistance can lose the ability to ambulate as they age. The decline is caused by increased body weight with growth without concurrent increased strength. By contrast, the patients who ambulate independently or with crutches can maintain the ambulation level as they age.

While 88% of the participants responded that they would recommend SDR, two percent would not recommend the procedure. None of the non-recommending patients were independently ambulating following SDR. This finding is expected since independently ambulating patients would have better perception of surgery. Nevertheless, it brings up the importance of clear communication and discussion between physicians and families about expected outcomes before surgery. Should a child be expected to walk with a walker after surgery in only protected environments, they should be aware that ambulation can decline with future growth.

Pain was reported in 38% of our patients and was not reported to be intense. The pain was mostly located in the back and the lower extremities. The pain problem in CP patients is well known. In a previous report on 88 adolescents and adults who underwent childhood SDR, chronic pain affected 51% [8]. In a study measuring the prevalence of pain in an overall CP adult population, 67% of 93 adults reported pain, which was often related to failed orthopedic surgeries [16]. In the general US population, 50% to 80% adults experience at least one episode of back pain during their lifetime [17].

Eleven percent of our patients reported urinary incontinence. None of the patients required intermittent catheterization. If urinary incontinence was due to SDR, perianal sensation would be absent. All the patients in our study had intact perianal sensation, suggesting that urinary incontinence could not have been a result of SDR intervention. On the other hand, urinary incontinence has been reported to affect 33% of CP patients [18]. The high incidence of urinary incontinence in the general CP population is likely due to uninhibited neurogenic bladder. In CP patients, the normal inhibitory control of the central nervous system detrusor function is impaired or underdeveloped, resulting in urgency or enuresis [19].

Diminished sensation in small areas of the lower extremities affected eight percent of the patients, though the sensory changes did not affect daily living. While 31% of patients reported a scoliosis diagnosis at the time of the survey, only three percent underwent spine fusion surgery. The reported incidence of scoliosis in all CP patients was 29% [20]. There is not enough evidence to suggest that scoliosis resulted from SDR in our patients and literature [21].

Utilizing a survey questionnaire to follow-up with SDR patients has made it possible for this study to collect long-term data on a large patient scale and to contribute unprecedented findings to the SDR literature. However, the use of a survey questionnaire as a primary research tool can present several limitations. Our study identified 431 eligible patients, though 115 patients could not be reached. It is possible that the patients who could not be reached did not have updated contact information because they did not return to our clinic for follow-up appointments. Therefore, the outcomes and quality of life in this patient population could be worse than reported in our study for various reasons. In addition, the survey data was collected in the form of subjective self-report questions that may present response bias. Our survey was also conducted through an online questionnaire, and the anonymity of responses may confound the exact breakdown of survey completion from patients to guardians.

## Conclusions

In our 95 adult patients who received SDR in childhood, the great majority of the patients reported positive surgical effects on the quality of life and ambulation 20-28 years later. In the patients participating in our study, there were no late complications of SDR surgery.
